# Waist Circumference, Not Body Mass Index, Is Associated with Renal Function Decline in Korean Population: Hallym Aging Study

**DOI:** 10.1371/journal.pone.0059071

**Published:** 2013-03-25

**Authors:** Hyunju Oh, Shan Ai Quan, Jin-Young Jeong, Soong-Nang Jang, Jung Eun Lee, Dong-Hyun Kim

**Affiliations:** 1 Department of Food and Nutrition, Sookmyung Women's University, Seoul, Korea; 2 Department of Social and Preventive Medicine, Hallym University, Chuncheon, Korea; 3 Korea Health Promotion Foundation, Seoul, Korea; 4 Department of Nursing, Chung-Ang University, Seoul, Korea; 5 Hallym Research Institute of Clinical Epidemiology, Chuncheon, Korea; University of Groningen, The Netherlands

## Abstract

**Background:**

Prospective investigation of obesity and renal function decline in Asia is sparse. We examined the associations of body mass index (BMI) and waist circumference (WC) with renal function decline in a prospective study of Korean population.

**Methods:**

A total of 454 participants who had baseline estimated glomerular filtration rate (eGFR) levels of more than 60 mL/min/1.73 m^2^ in Hallym Aging Study (HAS) were included and followed for 6 years. Renal function decline was defined as follows: (1) an eGFR decline ≥3 mL/min/1.73 m^2^/year (n = 82 cases); (2) an eGFR decrease of 20% or greater (n = 87 cases) at follow-up; (3) an eGFR decrease of 20% greater at follow-up or eGFR decline ≥3 mL/min/1.73 m^2^/year (n = 91 cases); and (4) an eGFR <60 mL/min/1.73 m^2^ at follow-up (n = 54 cases). eGFR was determined based on the Modification of Diet in Renal Disease (MDRD) Study equation. Multivariate logistic regression model was used to determine the association between obesity and renal function decline.

**Results:**

We found that central obesity was associated with faster renal function decline. Comparing WC of >95 cm in men or >90 cm in women with ≤90 cm in men or ≤85 cm in women, ORs (95% CIs) ranged from 2.31 (1.14–4.69) to 2.78 (1.19–6.50) for the 4 definitions of renal function decline (all p-values for trend <0.05). Waist-to-hip ratio (WHR) and waist-to-height ratio (WHtR) also was associated with renal function decline. There was no significant association of BMI with renal function decline.

**Conclusions:**

Central obesity, but not BMI, is associated with faster renal function decline in Korean population. Our results provide important evidence that simple measurement of central fat deposition rather than BMI could predict decline in renal function in Korean population.

## Introduction

Decreased kidney function is associated with a high risk of cardiovascular disease (CVD) [1]–[3], mortality [1], hospitalization [4], and low quality of life [5]. Chronic kidney disease (CKD) is one of the comorbid conditions of diabetes [6] and hypertension [2]. Glomerular filtration rate (GFR) and micro-/macroalbuminuria are often used as diagnostic markers for kidney function damage [7], [8]. GFR naturally decreases with aging by approximately 1 mL/minute/1.73 m^2^ per year [9], but either 3 or more mL/minute/1.73 m^2^/year of eGFR decline [10], [11] or low level of GFR (<60 mL/minute/1.73 m^2^) [8] reflects kidney dysfunction. The Kidney Disease Outcomes Quality Initiative (KDOQI) of the National Kidney Foundation (NKF) have defined CKD as a GFR of less than 60 mL/minute/1.73 m^2^ for more than 3 months [8]. GFR can be estimated through equations using circulating blood creatinine levels and demographic factors that affect filtration function [7], [12].

Obesity is a global health concern [13] and a major risk factor for diabetes [14], hypertension [15], and dyslipidemia [16], which may lead to decrease in renal function. Adipose tissue may affect the incidence and progression of CKD through release of inflammatory cytokines such as interleukin-6 or tumor necrosis factor-α [17]. Glomerular pressure caused by adipose tissues surrounding renal tissues may damage kidney function. Body mass index (BMI) has been the primary indicator for excess adipose tissue and consistently used in epidemiologic studies. However, there was suggestion that BMI could be limited to differentiate adipose tissue from lean body mass in intermediate BMI ranges, possibly less than 30 kg/m^2^ [18], [19]. The World Health Organization (WHO) recommends lower cutoff of BMI to define obesity for the Asia-Pacific region because Asians have smaller and slighter physique than Western population [20] and their health risks occur at a lower BMI [21]. Along with this evidence, we hypothesized that central obesity, reflected by waist circumference (WC), waist-to-hip ratio (WHR), waist-to-height ratio (WHtR), and fat distribution, rather than BMI could be a better predictor for chronic kidney disease. However, only a few Asian prospective studies examined central obesity in relation to eGFR decline [22]–[25].

Therefore, we examined the association of BMI and central obesity, as measured by WC, WHR, WHtR, with eGFR decline in the Hallym Aging Study (HAS), a prospective study of Korean population. We also compared four definitions of renal function decline (eGFR decline ≥3 mL/min/1.73 m^2^/year; eGFR decrease of 20% or greater at follow-up; eGFR decrease of 20% greater at follow-up or eGFR decline ≥3 mL/min/1.73 m^2^/year; and eGFR <60 mL/min/1.73 m^2^ at follow-up), their agreement, and their associations with obesity to identify a useful definition of renal function decline in the epidemiologic research of Korean population.

## Methods

### Study Design and Population

The HAS is a prospective cohort of 1,520 individuals (30% aged 45–64 years old and 70% aged 65 years old or greater) in Chuncheon, a small city in South Korea. Details of the HAS have been published elsewhere [26].

In brief, study populations were systematically sampled based on the Korean National Census conducted in 2000. One thousand, five hundred and twenty individuals were selected and completed panel surveys, while 918 participants among 1,520 agreed to participate in clinical examinations and completed face-to-face interviews at baseline in 2004. Two more follow-up examinations in 2007 and 2010 were performed. At each follow-up period, information on demographic factors, health behavior, and medical history, was obtained through face-to-face interviews using structured questionnaires. Blood and urine specimen were collected at each follow-up period in the morning following a minimum fasting period of 10 hours at Chuncheon Sacred Heart Hospital (80%) or at participants’ home (20%). Interviewers asked participants about their dietary habits using validated semi-quantitative food frequency questionnaire (FFQ)s [27] at baseline in 2004. Among 918 participants, 547 agreed to participate in 2007 follow-up examinations and 382 among 547 agreed to participate in 2010 follow-up examinations. Among those who had baseline creatinine levels (n = 840), 483 participants provided blood samples and had creatinine levels measured either in 2007 or 2010. We excluded participants if they had pre-existing of CKD (<60 mL/min/1.73 m^2^ of eGFR) at baseline (n = 32) and had inaccurate visit date (n = 1). Those participants with missing values for baseline anthropometric measurements were excluded from the analysis. Participants signed their own consent form and the ethics committee of Hallym University approved the research protocol.

### Assessments of Renal Function Decline

Serum creatinine levels at baseline and each visit were evaluated by the Jaffe method (HITACHI 7600-210, Hitachi, Japan). Coefficient of variance % was less than 3.5% for serum creatinine levels. Glomerular filtration rate was estimated based on the 4-variable Modification of Diet in Renal Disease (MDRD) Study equation as follows:

eGFR (mL/min/per 1.73 m^2^) = 186×(serum creatinine (mg/dL)^−1.154^)×(age^−0.203^)×(0.742 if female)×(1.210 if black) [12].

Also, we examined the associations between anthropometric measures and eGFR decline using the Chronic Kidney Disease Epidemiology Collaboration (CKD-EPI) equation [28] or Korean-specific eGFR equation [29] in the additional analyses.

The difference between the eGFR in 2004 and 2010 was calculated by subtracting the eGFR in 2010 from the eGFR in 2004. The158 participants, who did not have serum creatinine levels in 2010, eGFR in 2010 was calibrated based on the annual change in eGFR from 2004 to 2007. Among those with both baseline and 2010 measurements, the mean duration of the follow-up interval was 6.1±0.3 (standard deviation, SD) years. Annual change of eGFR and eGFR in 2010 was estimated as follows:

Annual change of eGFR (mL/min/1.73 m^2^/year) = (eGFR in either 2007 or 2010 − baseline eGFR)/(observational days/365 days).

eGFR in 2010 (mL/min/1.73 m^2^) = eGFR in 2007+ (annual change of eGFR×years from date of visit in 2007 to median date of visit in 2010).

We defined renal function decline as follows: (1) eGFR decline ≥3 mL/min/1.73 m^2^/year (n = 82 cases); (2) eGFR decrease of 20% or greater at follow-up (n = 87 cases); (3) eGFR decrease of 20% greater at follow-up or eGFR decline ≥3 mL/min/1.73 m^2^/year (n = 91 cases); and (4) eGFR <60 mL/min/1.73 m^2^ at follow-up (n = 54 cases). We compared the characteristics of study participants who revisited only in 2007 and those who revisited in both 2007 and 2010 using either a chi-square test or t-test (Table S1). Participants who completed the three visits had a slightly higher weight and were more educated than those who completed the two visits, whereas there was no difference in other factors.

### Anthropometric Measures

Weight to the nearest 0.01 kg and height to the nearest 0.1 cm were directly measured by health professional staff. For only a small proportion of participants, self-reported weight (1.8%) and height (0.7%) were obtained from the face-to-face interview. BMI was calculated as weight (kg) divided by squared height (m^2^). WC was measured at the midpoint between the lower costal margin and the level of the iliac crest. Hip circumference was measured at the most protruding part of the hips. WHR and WHtR were calculated by dividing WC (cm) by hip circumference (cm) or height (cm), respectively. Information on weight was available for all 454 participants. Measurements of height (n = 451), body fat (n = 412), and waist and hip circumference (n = 393) were not complete for some participants, therefore those who did not have information on these variables were excluded when each anthropometric variable was analyzed as the main exposure.

Body fat mass and body fat percent were measured by using bioelectrical impedance analysis (BIA, Inbody 3.0, Biospace, Korea). BMI was categorized into <23, 23 to <25, 25 to <30, and ≥30 kg/m^2^ in both men and women and WC was categorized into ≤90, 90 to ≤95, and >95 cm in men, and ≤85, 85 to ≤90, and >90 cm in women to pertain a certain number of cases. Other anthropometric measurements, including WHR, WHtR, body fat mass, and body fat percent, were grouped into tertiles.

### Assessment of Covariates

Blood pressures were obtained using average values from both arms at the beginning and end of the interview. Participants were considered to have a history of hypertension if they answered that they had been diagnosed with hypertension by a physician or if they had at baseline more than 140 mmHg of systolic blood pressure or more than 90 mmHg of diastolic blood pressure. Likewise, participants were considered to have a history of diabetes if they responded that they had been diagnosed with diabetes by a physician or if they had at baseline more than 126 mg/dl of fasting blood sugar levels. Pack-years of smoking were calculated by number of cigarettes smoked and number of years smoked. Duration of education was categorized into 3 groups by years of education: none, 1 to <6 years, and ≥6 years. Total energy intake was calculated from the baseline semi-quantitative FFQs.

### Statistical Analysis

We compared the characteristics of 4 definitions of renal function decline by presenting the percentage for categorical data and the means ± SD for continuous data. Comparison of characteristics was performed by chi-square test for categorical variables or by t-test for continuous variables. Multiple logistic regression analyses were used to estimate odds ratio (OR) and 95% confidence intervals (CI) for the association between anthropometric measurements and renal function decline adjusted for age, sex, baseline eGFR, total energy intake, duration of education, pack-years of smoking, and history of hypertension and diabetes. We adjusted for duration of education because we found that those with renal function decline were more likely to have lower duration of education compared to non-cases. The median value for each category or tertile of exposure was used to examine a trend of the association. Stratified analyses according to sex, age (young, <69 years; older, ≥69 years), history of chronic disease (hypertension, diabetes, cardiovascular disease, myocardial infarction or other heart disease; never, at least 1), smoking status (never, ever), and duration of education (≤6 years, >6 years) were conducted to examine the interactions. Heterogeneity for interactions was tested by creating a cross-product term of the eGFR decline for each level of stratified factor and obesity factors. Statistical tests were 2-sided, and a P-value <0.05 was considered statistically significant. All analyses were performed using the SAS (version 9.2, Cary, NC, USA).

## Results

Baseline characteristics of all participants and cases are shown in Table 1 according to the 4 definitions of renal function decline. Of all participants, 11.9% developed renal function decline whose eGFR dropped below 60 mL/min/1.73 m^2^ at follow-up, while 18.1–20% of participants fell into the other three definitions of renal function decline. The mean (SD) age was 67.4 (8.4) years (27.5% were 46–64 years old and 72.5% were 65–85 years old). The mean (SD) serum creatinine level was 0.8 (0.2) mg/dl, and the mean (SD) eGFR was 84.5 (14.5) mL/min/1.73 m^2^ among all participants. The baseline mean eGFR value was higher among cases of renal function decline than all participants in the study, but individuals whose eGFR was <60 mL/min/1.73 m^2^ at follow-up had a lower baseline eGFR when compared to all participants in the study. Baseline WC and weight were slightly higher for cases with eGFR <60 mL/min/1.73 m^2^ at follow-up than the other three definition cases of renal function decline. Other baseline anthropometric measures were similar across the four case definitions. The proportions of baseline history of diabetes or hypertension were higher among all four case definitions of renal function decline when compared to overall participants in the study, with the highest proportion found among cases with eGFR <60 mL/min/1.73 m^2^ at follow-up. All low eGFR cases were less likely to currently smoke and attend school for more than 6 years when compared to all participants in the study.

**Table 1:** Baseline characteristic of participants and cases according to renal function decline in the HAS in Korea, 2004.

	Overall (n = 454)	≥3 decline (n = 82)	20% decline (n = 87)	≥3 or 20% decline (n = 91)	60> eGFR (n = 54)
Age, years	67.4±8.4	67.8±9.2	69.0±8.7	68.5±9.1	71.4±7.1
Creatinine, mg/dL	0.8±0.2	0.8±0.1	0.8±0.2	0.8±0.2	0.9±0.2
eGFR, mL/min/1.73 m^2^	84.5±14.5	94.2±17.2	91.1±17.8	91.9±17.9	81.4±15.5
BMI, kg/m^2^	25.0±3.3	25.2±3.8	25.2±3.7	25.2±3.7	25.6±3.3
WC, cm	86.4±8.7	88.3±9.2	88.8±8.9	88.5±9.0	90.3±7.9
Weight, kg	60.6±10.2	59.6±10.3	60.3±10.4	60.3±10.4	61.6±10.8
Height, cm	155.5±9.6	154.0±10.0	154.8±9.9	154.7±9.9	155.3±9.6
Hip circumference, cm	93.8±6.2	95.2±5.8	94.9±5.9	94.8±5.9	94.3±5.8
WHR	0.9±0.1	0.9±0.1	0.9±0.1	0.9±0.1	1.0±0.1
WHtR	0.6±0.1	0.6±0.1	0.6±0.1	0.6±0.1	0.6±0.1
Body fat mass, kg	18.1±5.7	19.2±6.3	19.2±6.1	19.2±6.2	19.2±5.7
Body fat percent, %	29.6±7.4	31.5±8.1	31.3±7.8	31.2±7.9	30.8±7.1
Male (%)	43.0	34.2	40.2	38.5	46.3
Diabetes (%)	13.0	15.9	17.2	17.6	22.2
Hypertension (%)	54.9	61.0	63.2	63.7	72.2
Smoking (%)					
Never	61.5	67.1	62.1	63.7	57.4
Past	24.2	20.7	25.3	24.2	31.5
Current	14.3	12.2	12.6	12.1	11.1
Education duration (%)					
Never	25.1	34.2	32.2	33.0	38.9
≤6 years	43.4	40.2	42.5	40.7	35.2
>6 years	31.5	25.6	25.3	26.4	25.9

Note: Continuous variables are presented by mean±SD (standard deviation); Some variables had missing values for overall and 4 cases.

Abbreviations: HAS, Hallym aging study; eGFR, estimated glomerular filtration rate; BMI, body mass index; WC, waist circumference; WHR, waist-to-hip ratio; and WHtR, waist-to-height.ratio.

Table 2 shows concordance and discordance among the four definitions of renal function decline according to eGFR decline. Concordance rates were 97% between more than 20% decline of eGFR over 6 years and ≥3 mL/min/1.73 m^2^/year of eGFR decline, and 88–91% between eGFR <60 mL/min/1.73 m^2^ at follow-up and the other three categories of renal function decline. Proportions of concordance by CKD-EPI equation or equation developed for Korean were similar with the results estimated by MDRD equation (data not shown).

**Table 2:** Concordant and discordant occurrence of eGFR decline.

		20% decline		≥3 or 20% decline		60> eGFR	
		No	Yes	No	Yes	No	Yes
≥3 decline	No	363	9	363	9	359	13
	Yes	4	78	0	82	41	41
20% decline	No	–	–	363	4	363	4
	Yes	–	–	0	87	37	50
≥3 or 20% decline	No	–	–	–	–	359	4
	Yes	–	–	–	–	41	50

Abbreviations: eGFR, estimated glomerular filtration rate.

We found that central obesity was associated with faster renal function decline in Korean population in a multivariate model adjusted for potential confounding factors (all p-values for trend <0.02) (Table 3). Similarly, high WC levels associated with renal function decline estimated by CKD-EPI equation or by equation developed for Korean. For CKD-EPI equation, ORs (95% CIs) ranged from 2.29 (1.08–4.86) to 3.01(1.34–6.79) comparing WC of >95 cm in men or >90 cm in women with ≤90 cm in men or ≤85 cm in women. When we used equation developed for Korean, ORs (95% CIs) ranged from 2.79 (1.29–6.02) to 3.12 (1.53–6.35) comparing WC of >95 cm in men or >90 cm in women with ≤90 cm in men or ≤85 cm in women. The associations between the tertiles of WC and eGFR decline were statistically significant for all of 4 definitions of renal function decline (all p-values for trend <0.02). Compared to the lowest WC tertile, those in the highest WC tertile had ORs (95% CIs) of 2.47 (1.19–5.15) to 3.83 (1.44–10.19) across the different definitions of renal function decline. When we adjusted for BMI in the analysis of WC, the association for WC became de-attenuated (OR = 3.17; 95% CI, 1.10–9.13; p value for trend = 0.02 when comparing WC of >95 cm in men or >90 cm in women with ≤90 cm in men or ≤85 cm in women). When WC was analyzed as a continuous variable, a 1 cm increase in WC was associated with a statistically significant a 5% increase in risk of having an eGFR <60 mL/min/1.73 m^2^ at follow-up (OR = 1.05, 95% CI, 1.01–1.10, p-value = 0.02). After further adjustment for hip circumference, a 1 cm increase in WC was associated with a 7% increase in risk of having an eGFR <60 mL/min/1.73 m^2^ at follow-up (OR = 1.07, 95% CI, 1.02–1.13, p-value = 0.01). WHR and WHtR also generally were associated with occurrence of eGFR <60 mL/min/1.73 m^2^. However, their magnitude and statistical significance varied across case definitions with the strongest association for cases of eGFR <60 mL/min/1.73 m^2^.

**Table 3:** Multivariate odds ratio (ORs) and 95% confidence intervals (CIs) of eGFR decline according to tertiles or categories of anthropometric measures.

Measures	Category/Tertile	≥3 decline		20% decline		≥3 or 20% decline		60> eGFR	
		No. of case	OR (95% CI)	No. of case	OR (95% CI)	No. of case	OR (95% CI)	No. of case	OR (95% CI)
BMI^a^ (kg/m^2^)	<23	20	1.00	21	1.00	22	1.00	12	1.00
	23−<25	20	1.13(0.53–2.42)	23	1.24(0.61–2.54)	23	1.16(0.57–2.37)	12	1.08(0.43–2.67)
	25−<30	30	1.05(0.51–2.17)	31	1.01(0.51–2.01)	33	1.00(0.51–1.98)	22	1.24(0.54–2.84)
	≥30	10	2.15(0.80–5.77)	10	2.14(0.82–5.58)	11	2.21(0.86–5.67)	6	2.12(0.67–6.76)
	P-trend		0.3		0.3		0.3		0.2
BMI^b^ (kg/m^2^)	<23	20	1.00	21	1.00	22	1.00	12	1.00
	23−<25	20	0.85(0.38–1.93)	23	0.90(0.42–1.94)	23	0.88(0.41–1.89)	12	0.75(0.29–1.97)
	25−<30	30	0.62(0.24–1.55)	31	0.54(0.23–1.31)	33	0.59(0.25–1.40)	22	0.59(0.21–1.69)
	≥30	10	0.96(0.26–3.57)	10	0.85(0.24–2.98)	11	0.99(0.29–3.43)	6	0.70(0.16–3.16)
	P-trend		0.7		0.5		0.7		0.5
WC^a^ (cm)	M: < = 90, F: < = 85	23	1.00	27	1.00	28	1.00	16	1.00
	M: >90, F: >85	18	2.63(1.22–5.65)	17	2.00(0.96–4.16)	18	2.00(0.97–4.13)	11	2.80(1.14–6.92)
	M: >95, F: >90	21	2.49(1.15–5.38)	24	2.38(1.17–4.83)	25	2.35(1.16–4.74)	17	2.84(1.22–6.61)
	P-trend		0.01		0.01		0.01		0.01
WC^c^ (cm)	M: < = 90, F: < = 85	23	1.00	27	1.00	28	1.00	16	1.00
	M: >90, F: >85	18	2.98(1.26–7.02)	17	2.11(0.94–4.74)	18	2.24(1.00–5.01)	11	2.88(1.06–7.79)
	M: >95, F: >90	21	3.17(1.10–9.13)	24	2.65(1.00–6.99)	25	2.93(1.12–7.69)	17	2.98(0.94–9.48)
	P-trend		0.02		0.04		0.02		0.05
Weight^d^ (cm)	T1	31	1.00	30	1.00	32	1.00	16	1.00
	T2	25	0.96(0.48–1.92)	28	1.13(0.58–2.20)	29	1.05(0.54–2.03)	17	1.36(0.58–3.21)
	T3	26	1.30(0.58–2.95)	29	1.40(0.64–3.10)	30	1.28(0.59–2.80)	21	2.09(0.76–5.75)
	P-trend		0.6		0.4		0.5		0.2
Height^e^ (cm)	T1	30	1.00	29	1.00	31	1.00	16	1.00
	T2	25	1.13(0.54–2.35)	27	1.21(0.59–2.48)	28	1.22(0.60–2.48)	14	1.53(0.59–3.93)
	T3	25	1.84(0.58–5.84)	29	1.62(0.54–4.85)	30	1.82(0.61–5.43)	22	3.87(0.91–16.5)
	P-trend		0.3		0.4		0.3		0.06
Hip circumference^a^ (cm)	T1	13	1.00	15	1.00	16	1.00	11	1.00
	T2	25	2.29(1.04–5.03)	28	2.24(1.08–4.67)	29	2.10(1.02–4.31)	19	2.16(0.90–5.14)
	T3	24	1.95(0.87–4.38)	25	1.88(0.88–3.99)	26	1.70(0.81–3.57)	14	1.56(0.63–3.89)
	P-trend		0.1		0.1		0.2		0.4
WHR^a^	T1	21	1.00	21	1.00	22	1.00	7	1.00
	T2	13	0.52(0.23–1.19)	13	0.49(0.22–1.08)	15	0.56(0.26–1.20)	9	1.15(0.40–3.35)
	T3	28	1.64(0.80–3.35)	34	1.76(0.90–3.43)	34	1.74(0.89–3.39)	28	3.94(1.55–9.97)
	P-trend		0.1		0.04		0.05		0.001
WHtR^a^	T1	16	1.00	20	1.00	21	1.00	10	1.00
	T2	14	0.86(0.38–1.98)	14	0.62(0.29–1.35)	14	0.58(0.27–1.27)	9	0.85(0.32–2.29)
	T3	32	2.25(1.02–4.97)	34	1.76(0.85–3.64)	36	1.79(0.88–3.67)	25	3.04(1.22–7.61)
	P-trend		0.03		0.08		0.07		0.01
Body fat mass^a^ (kg)	T1	22	1.00	24	1.00	25	1.00	13	1.00
	T2	17	0.69(0.33–1.48)	19	0.75(0.37–1.53)	19	0.70(0.34–1.42)	15	1.51(0.65–3.55)
	T3	28	1.20(0.57–2.54)	30	1.27(0.63–2.58)	32	1.29(0.64–2.59)	20	2.07(0.87–4.95)
	P-trend		0.5		0.4		0.4		0.1
Body fat percent^a^ (%)	T1	18	1.00	20	1.00	21	1.00	13	1.00
	T2	20	1.08(0.49–2.39)	23	1.22(0.58–2.56)	23	1.15(0.55–2.39)	18	1.31(0.56–3.07)
	T3	29	1.25(0.51–3.09)	30	1.47(0.62–3.50)	32	1.46(0.62–3.42)	17	1.43(0.50–4.13)
	P-trend		0.6		0.4		0.4		0.5

Abbreviations: OR, odds ratio; CI, confidence interval; M, male; F, female; eGFR, estimated glomerular filtration rate; BMI, body mass index; WC, waist circumference.

a Adjusted for age (continuous), sex, baseline eGFR (continuous), pack-years of smoking (continuous), duration of education (none, 1 to <6 years, ≥6 years), total energy intake (continuous), and history of hypertension or diabetes (yes, no).

b Adjusted for variables above and WC (continuous).

c Adjusted for variables above and BMI (continuous).

d Adjusted for variables above and height (continuous).

e Adjusted for variables above and weight (continuous).

We found no statistical significant associations of eGFR decline with high BMI in multivariate models. Simultaneous adjustment for WC attenuated the association with BMI. For example, OR (95% CI) was 0.70 (0.16–3.16) for cases of eGFR <60 mL/min/1.73 m^2^ when comparing more than 30 kg/m^2^ with <23 kg/m^2^ of BMI. Similarly, there were no statistical associations of BMI levels with renal function decline when we used CKD-EPI equation or equation developed for Korean. For hip circumference, faster renal function decline was observed in the second tertile. Weight and height were not significantly associated with renal function decline. Body fat mass and body fat percent did not show significant associations with renal function decline.

When we examined whether sex, age, smoking status, duration of education, and history of diabetes or hypertension modified the associations between BMI or WC and occurrence of eGFR <60 mL/min/1.73 m^2^ at follow-up, we did not find any statistically significant interactions for these factors (P values for interaction >0.06; data not shown).

## Discussion

In this study, we observed that central obesity was associated with faster renal function decline, as defined by a decline in eGFR, in a community-based Korean population. High levels of WC, WHR, and WHtR were significantly associated with faster eGFR decline. BMI, body fat mass or body fat percent were not associated with renal function decline, suggesting that central deposition of fat may be a more important factor for renal function decline than total body fat mass in Korean populations.

When we examined these associations for 4 different definitions of renal function decline (eGFR decline ≥3 mL/min/1.73 m^2^/year; eGFR decrease of 20% or greater at follow-up; eGFR decrease of 20% greater at follow-up or eGFR decline ≥3 mL/min/1.73 m^2^/year; and eGFR <60 mL/min/1.73 m^2^ at follow-up), the magnitude of the associations for abdominal measurements varied across the definitions, but the significance and associations were more clearly observed for those participants whose eGFR levels were less than 60 mL/min/1.73 m^2^. Previous studies demonstrated that eGFR decline was a good marker for kidney dysfunction [7]. CKD is diagnosed through GFR measurement and the presence of kidney damage identified by abnormalities in the composition of markers, such as albumin in urine, and pathological abnormalities in imaging tests. However, these diagnostic methods make it challenging to find meaningful criteria of renal dysfunction in large epidemiologic studies. Various definitions of renal function decline have been employed across epidemiologic studies to identify associated risk factors [1], [30]–[32], but only a few attempted to determine a standard definition for prospective study research [30], [31].

Several epidemiologic studies, mainly conducted in Western countries, suggest that BMI [11], [32], [33] is a strong risk factor for CKD, as well as central obesity [11], [33], [34]. A few Asian prospective studies including Korea [35], Japan,[36]–[38] and China [25], have investigated the association of BMI with CKD risk and have yielded inconsistent results. In Japanese cohort studies [36]–[38], BMI was associated with reduced eGFR, whereas Korean and Chinese studies found no [35], [39], [40] or inverse [25] association for BMI in relation with eGFR decline. For central obesity, although several Asian cross-sectional studies observed positive associations between WC and the prevalence of CKD [41]–[46], these studies were limited because of lack of temporal relationships inherent in a cross-sectional design. Only a few Asian prospective studies examined WC and incidence of CKD. In Southeast Asian cohort study followed for 12 years, WC was not significantly associated with risk of incident CKD [22]. A recent Japanese cohort study that examined the changes in eGFR levels 1 year apart found that participants with increased WC group had rapid decline of eGFR (−6.0±10.2mL/min/1.73 m^2^) compared to those with decreased WC group (−2.7±8.2mL/min/1.73 m^2^) [23]. In Hong Kong study, central obesity increased risk for CKD among patients with diabetes, reporting hazard ratio (HR) of 1.58 (1.26–1.98) comparing high WC level (>80 cm in women and >90 cm in men) with low WC level, whereas those with BMI>25kg/m^2^ had a lower risk of CKD compared to those with low BMI [25]. Also, Taiwanese cohort study found higher risk of CKD with increased WC [24].

A mechanism relating obesity to renal damage may involve inflammation, oxidative stress, and insulin resistance and pathophysiology alteration. Adipose tissue may affect the incidence and progression of CKD through the release of inflammatory cytokines such as interleukin-6 or tumor necrosis factor-α [17]. Adiposity may amplify inflammation and oxidative stress [47], both of which may contribute to kidney damage by a proinflammatory mechanism [48]. Fat mass also aggravates insulin resistance, which may be related to kidney damage [49]. Glomerular pressure caused by adipose tissues surrounding renal tissues may cause damage to kidney function. According to the Framingham Heart Study, kidney sinus fat was associated with an increased risk for CKD (P = 0.04) even after adjusting for BMI [50]. Because central obesity is a strong risk factor for diabetes [14], hypertension [15] or CVD [51], and all of these diseases increase the risk of CKD [2], [6], a high susceptibility to diabetes, hypertension or CVD among those with abdominal fat mass could lead to the development of CKD. However, faster renal function decline with increased central obesity was still observed even after adjusting for a history of diabetes or hypertension in our study, suggesting that adiposity may be directly related to the development of renal damage independent of hypertension or diabetes.

Although we found that no association of BMI with renal function decline, WC, WHR, and WHtR appeared to be strong risk factors for the development of renal function decline. Previous studies suggested that central obesity, often measured by WC or WHR, is a better predictor than BMI to reflect visceral fat mass [52], or predict some chronic diseases [34], [53] and mortality [34], [52], [54] in elderly populations. BMI may not fully reflect the fat mass and distribution in elderly populations [52] because the reduced muscle mass in the elderly could also lower the overall body mass, and a high BMI does not necessarily indicate high visceral fat mass. This observation is also supported by several epidemiologic studies. In both the Atherosclerosis Risk in Communities Study and the Cardiovascular Health Study, WHR, but not BMI, was associated with an increased risk of CKD and mortality in 13,324 men and women [34]. When WC was examined within similar BMI levels or with adjustment for BMI in cohort [53], [54] and cross-sectional studies [15], WC was independently associated with the risk of mortality [54] and coronary heart disease [53], as well as the prevalence of metabolic syndrome [15].

Our study has several limitations. First, our study population may not represent the population in Korea because study participants resided in a small city in Korea. Second, the eGFR value of 158 participants (whose demographics and serum creatinine levels were not assessed in 2010) were estimated using the change rate per year from 2004 to 2007. However, when we observed characteristics between those who revisited in 2007 and those who revisited in 2007 and 2010, participants had similar characteristics in the majority of factors examined at baseline. Third, there can be some degree of measurement error in anthropometric measures, but WC and hip circumference were all directly measured, and height and weight were directly measured in almost all participants (>98%) in our study.

The strengths of our study included a prospective study with 6 years of follow-up and the repeated measurement of biomarkers which allowed for the estimation of change in eGFR. To our knowledge, this is the first prospective study that has examined the associations of WC, WHR, and WHtR with renal function decline Korean population.

In conclusion, our data supports the evidence that central obesity may be a better predictor for renal function decline than BMI in Korean population. A reduction in central obesity should be emphasized as a key component for the prevention of renal function decline. Further studies in Korean populations are warranted to confirm our finding.

## Supporting Information

Table S1 **Comparison in eGFR between participants revisit only in 2007 and those both in 2007 and 2010.**
(XLS)
